# Intratracheal trimerized nanobody cocktail administration suppresses weight loss and prolongs survival of SARS-CoV-2 infected mice

**DOI:** 10.1038/s43856-022-00213-5

**Published:** 2022-11-26

**Authors:** Kayoko Nagata, Daichi Utsumi, Masamitsu N. Asaka, Ryota Maeda, Kotaro Shirakawa, Yasuhiro Kazuma, Ryosuke Nomura, Yoshihito Horisawa, Yohei Yanagida, Yugo Kawai, Kei Sato, Yutaro Yamaoka, Kei Miyakawa, Akihide Ryo, Yasuhiro Yasutomi, Akihiro Imura, Akifumi Takaori-Kondo

**Affiliations:** 1 Department of Hematology and Oncology, Graduate School of Medicine, Kyoto University, Kyoto, 606-8507 Japan; 2 Tsukuba Primate Research Center, National Institutes of Biomedical Innovation, Health and Nutrition, Tsukuba, Ibaraki 305-0843 Japan; 3 COGNANO Inc., Kyoto, 601-1255 Japan; 4 Division of Systems Virology, Department of Microbiology and Immunology, The Institute of Medical Science, The University of Tokyo, Tokyo, Japan; 5 International Research Center for Infectious Diseases, The Institute of Medical Science, The University of Tokyo, Tokyo, Japan; 6 International Vaccine Design Center, The Institute of Medical Science, The University of Tokyo, Tokyo, Japan; 7 Graduate School of Medicine, The University of Tokyo, Tokyo, Japan; 8 CREST, Japan Science and Technology Agency, Saitama, Japan; 9 Department of Microbiology and Molecular Biodefense Research, Yokohama City University Graduate School of Medicine, Yokohama, 236-0004 Japan

**Keywords:** SARS-CoV-2, Antiviral agents, Viral infection, Antibody generation, Drug development

## Abstract

**Background:**

SARS-CoV-2 Omicron variants are highly resistant to vaccine-induced immunity and human monoclonal antibodies.

**Methods:**

We previously reported that two nanobodies, P17 and P86, potently neutralize SARS-CoV-2 VOCs. In this study, we modified these nanobodies into trimers, called TP17 and TP86 and tested their neutralization activities against Omicron BA.1 and subvariant BA.2 using pseudovirus assays. Next, we used TP17 and TP86 nanobody cocktail to treat ACE2 transgenic mice infected with lethal dose of SARS-CoV-2 strains, original, Delta and Omicron BA.1.

**Results:**

Here, we demonstrate that a novel nanobody TP86 potently neutralizes both BA.1 and BA.2 Omicron variants, and that the TP17 and TP86 nanobody cocktail broadly neutralizes in vitro all VOCs as well as original strain. Furthermore, intratracheal administration of this nanobody cocktail suppresses weight loss and prolongs survival of human ACE2 transgenic mice infected with SARS-CoV-2 strains, original, Delta and Omicron BA.1.

**Conclusions:**

Intratracheal trimerized nanobody cocktail administration suppresses weight loss and prolongs survival of SARS-CoV-2 infected mice.

## Plain language summary

Antibodies are made by the immune system to identify and inactivate infectious agents such as viruses. Alpacas produce a simple type of antibodies called nanobodies. We previously developed two nanobodies named P17 and P86 that inactivate SARS-CoV-2. In this study, we modified these nanobodies to create two nanobodies named TP17 and TP86. The cocktail of these nanobodies inactivated different types of SARS-CoV-2 viruses including Omicron BA.1 and BA.2. The cocktail also prolonged survival of mice infected with lethal doses of SARS-CoV-2.

## Abstract (web summary)

Nagata, Utsumi, Asaka, Maeda et al. describe a nanobody, TP86, that potently neutralizes both BA.1 and BA.2 Omicron SARS-CoV-2 variants, and, when combined with the TP17 nanobody, broadly neutralizes all SARS-CoV-2 variants. This nanobody cocktail also suppresses weight loss and prolongs survival of SARS-CoV-2 infected human ACE2 transgenic mice.

## Introduction

The emergence of a new severe acute respiratory syndrome coronavirus 2 (SARS-CoV-2) variant, Omicron, is now a threat to world health. The Omicron (B.1.1.529) variant was first reported in South Africa and classified as the 5th variant of concern (VOC) in November 2021^1^. The original form, BA.1, spread rapidly and became dominant worldwide, outcompeting variant Delta. Continuous surveillance revealed that BA.1 was outcompeted by the new sublineage BA.2. The Omicron variants have more than 30 mutations in the spike protein and evade immune response, being highly resistant to vaccine-induced humoral immunity and human monoclonal antibody drugs^2–5^. Recent studies have revealed that BA.1 is less fusogenic and less pathogenic than the original strain^6,7^, whereas BA.2 is more infectious and pathogenic than BA.1^8^.

A nanobody is the 15-kDa antigen-binding fragment of the camelid antibody which consists of only the heavy chain^9^. Nanobodies show unique characteristics such as: stable, highly soluble, small enough to access narrow spaces, usable as aerosols, and easy to produce and modify^10^. After the start of the COVID-19 pandemic, many nanobodies against SARS-CoV-2 have been produced and tested^11^. However, their efficacy against Omicron remains unclear.

We previously reported that two nanobodies, P17 and P86, potently neutralize SARS-CoV-2 VOCs including Alpha/B.1.1.7, Beta/B.1.351, Gamma/P.1, Delta/B.1.617.2, and Omicron/BA.1/BA.2^12,13^. We also determined the epitopes recognized by these nanobodies (P17 and P86) using Cryo-EM. The epitopes were located on the outer side of RBD; even outer than that recognized by previously reported class 3 human neutralizing Abs. It seems that VHH is small enough to access the hidden clef that is not recognized by human neutralizing Abs^12,14^.

In this study, we modify P17 and P86 into trimers named TP16 and TP86 respectively and show that the cocktail of these nanobodies neutralizes SARS-CoV-2 VOCs using in vitro pseudoviral infectivity assays and that the cocktail prolongs survival of mice infected with lethal doses of SARS-CoV-2 in vivo.

## Materials and methods

### Nanobody production and trimerization

Nanobodies targeting SARS-CoV-2 spike protein were selected as previously described^12,13^. Genes of N-terminally PelB (MKYLLPTAAAGLLLLAAQPAMA)-tagged tandem homotrimer of the nanobody connected with two (GGGGS)_4_ linkers and C-terminally 6×His-tagged were synthesized and subcloned in the pMES4 vector. The exact amino acid sequences are as below:

TP17, MKYLLPTAAAGLLLLAAQPAMAQVQLQESGGGLVQAGGSLRLSCAASGRTSSVYNMAWFRQTPGKEREFVAAITGNGGTTLYADSVKGRLTISRGNAKNTVSLQMNVLKPDDTAVYYCAAGGWGKERNYAYWGQGTQVTVSSGGGGSGGGGSGGGGSGGGGSQVQLQESGGGLVQAGGSLRLSCAASGRTSSVYNMAWFRQTPGKEREFVAAITGNGGTTLYADSVKGRLTISRGNAKNTVSLQMNVLKPDDTAVYYCAAGGWGKERNYAYWGQGTQVTVSSGGGGSGGGGSGGGGSGGGGSQVQLQESGGGLVQAGGSLRLSCAASGRTSSVYNMAWFRQTPGKEREFVAAITGNGGTTLYADSVKGRLTISRGNAKNTVSLQMNVLKPDDTAVYYCAAGGWGKERNYAYWGQGTQVTVSSHHHHHH;

TP86, MKYLLPTAAAGLLLLAAQPAMAMAQVQLQESGGGLVQAGGSLRLSCVASGRTFSSLNIVWFRQAPGKERKFVAAINDRNTAYAESVKGRFTISRDNAKNTVHLQMNSLKPEDTAVYYCHSADVNGGMDYWGKGTQVTVSSGGGGSGGGGSGGGGSGGGGSQVQLQESGGGLVQAGGSLRLSCVASGRTFSSLNIVWFRQAPGKERKFVAAINDRNTAYAESVKGRFTISRDNAKNTVHLQMNSLKPEDTAVYYCHSADVNGGMDYWGKGTQVTVSSGGGGSGGGGSGGGGSGGGGSQVQLQESGGGLVQAGGSLRLSCVASGRTFSSLNIVWFRQAPGKERKFVAAINDRNTAYAESVKGRFTISRDNAKNTVHLQMNSLKPEDTAVYYCHSADVNGGMDYWGKGTQVTVSSHHHHHH.

These expression vectors were introduced in the lipopolysaccharide-free electrocompetent BL21 (DE3) *E. coli* according to the manufacturer’s protocol (ClearColi: LGC Ltd., Middlesex, UK). The transformed colonies were selected and grown in the phosphate buffered broth. When the *E. coli* culture broth reached an OD of 0.6 AU, the final concentrations of 1 mM isopropyl-β-D-thiogalactopyranoside was added to the cells and the cells were continued to culture for several hours. The cultured *E. coli* cells were collected with centrifugation (2100 × *g*, 4 °C for 10 min) and suspended with the TES buffer containing 200 mM Tris (pH 8.0), 0.5 mM EDTA, and 500 mM sucrose. After incubating the cells at 4 °C for 1 h, 2× volumes of a diluted TES buffer containing 50 mM Tris (pH 8.0), 0.125 mM EDTA, and 125 mM sucrose was added and the cells were further incubated at 4 °C for 45 min, and the supernatants were centrifuged (20,000 × *g*, 4 °C for 10 min) and collected. The extracted nanobodies were purified using IMAC (Cytiva) and desalted with a dialysis membrane.

### Cell lines

LentiX-HEK293T cells (Takara Bio #Z2180N) were maintained in DMEM (high glucose) (Sigma-Aldrich, #6429) containing 10% fetal bovine serum (FBS, Sigma-Aldrich #172012), and 1% penicillin-streptomycin (PS) (Sigma-Aldrich, #P4333). HOS cells stably express human ACE2 and TMPRSS2 (HOS-ACE2-TMPRSS2 cells) were prepared as previously described^15^. VeroE6/TMPRSS2 cells were obtained from the JCRB Cell Bank of NIBIOHN for SARS-CoV-2 virion preparation.

### Pseudoviral infectivity assay

HIV-1-based SARS-CoV-2 spike pseudotyped virus was prepared as described previously^12,13^. In brief, LentiX-HEK293T cells were transfected with plasmids encoding the C-terminally C9-tagged full-length SARS-CoV-2 spike variants (D614G, Beta, Gamma, Delta, and Omicron) and HIV-1 transfer vector encoding a luciferase reporter using PEI MAX transfection reagent (Polyscience #24765). Cells were incubated for 3.5 h at 37 °C with DNA/PEI mixture and with DMEM containing 10% FBS for another 48 h. The supernatants were then collected, filtered through a 0.45-mm membrane, and centrifuged. The pseudoviruses were incubated with four-fold sequentially diluted nanobodies for 1 h at 37 °C. As control, pseudoviruses were also incubated without nanobodies. Then, the pseudoviruses with and without nanobodies were added onto HOS-ACE2-TMPRSS2 cells and cultured for 2 days. The infected cells were lysed, and luciferase activity was measured using the Bright-Glo Luciferase Assay System (Promega KK, Osaka, Japan) with a microplate spectrophotometer (ARVO X3: PerkinElmer Japan Co., Ltd., Kanagawa, Japan). All assays were performed in triplicate and IC_50_ values were calculated using the GraphPad Prism software. Original data are available in Supplementary Data.

### Preparation of SARS-CoV-2 virions

Tokyo strain (SARS-CoV-2/UT-NCGM02/Human/2020/Tokyo) and Omicron strain (hCoV-19/Japan/NC928-2N/2021) were provided by National Center for Global Health and Medicine. Delta strain (TKYTK1734) was provided by Tokyo Metropolitan Institute of Public Health. Tokyo strain and Delta strain were infected with VeroE6/TMPRSS2 at an MOI of 0.1 and then cultured in DMEM containing 2% FBS at 37 °C for 1 day. Omicron strain was infected with VeroE6/TMPRSS2 at an MOI of 0.1 and then cultured in DMEM containing 2% FBS at 37 °C for 3 days. The culture media were centrifuged at 1500 × *g* for 10 min, then stored at –80 °C. To measure the viral titer, culture media were diluted serially by a factor of 10 with RPMI1640 containing 2% FBS and PS. The diluted culture media were incubated with VeroE6/TMRPSS2 cells (2 × 10^4^ cells/well) in 96 well plates for 3 to 5 days, and viral titers of each strain were calculated using the Reed-Muench calculation method.

### In vivo infection assay using huACE2 transgenic mice

huACE2 mice were obtained from the Laboratory Animal Resource Bank of the National Institute of Biomedical Innovation, Health and Nutrition. To maintain the heterozygous huACE2 mice, C57BL/6 mice and heterozygous huACE2 mice were mated. The genotypes of mice were analyzed by PCR for ear DNA using the primer sets 5′- CTTGGTGATATGTGGGGTAGA -3′ and 5′- CGCTTCATCTCCCACCACTT -3′. Male and female huACE2 mice were maintained in plastic cages with free access to food and water and housed at 25 ± 2 °C with a 12 h light/dark cycle. huACE2 Tg mice were assigned randomly to two groups (PBS-treatment (*n* = 6) and TP17/86 cocktail-treatment (*n* = 6)) to assess the protective efficacy of the TP17/86 cocktail. huACE2 Tg mice were inserted intubation tube (22G 32 mm, KN-1008-2, Natsume Seisakusho) using an otoscope and intubation platform under anesthesia (100 μl/mouse, Medetomidine: 20 μg/ml, Midazolam: 600 μg/ml, Butorphanol: 1 mg/ml) and then infected via respiratory tract with SARS-CoV-2 virus (ancestral and Delta, at a dosage of 1 × 10^4^ TCID_50_ in 25 μl; Omicron at a dosage of 1 × 10^5^ TCID_50_ in 25 μl) using a 100 μl micropipette. Infected mice were intraperitoneally injected with atipamezole (100 μl/mouse, 20 μg/ml). One day post infection, infected mice received intratracheally 1.2 mg/kg of TP17/86 cocktail (VHH) or PBS (Control) similar to how infection was performed. Body weight and survival of the infected mice were monitored every day for up to 14 days. Mice that were clearly emaciated were euthanized after recording their body weight and were considered dead. Original data are available in Supplementary Data.

### Ethics statements

All mice experiments were performed in accordance with the Science Council of Japan’s Guidelines for the Proper Conduct of Animal Experiments. The protocols were approved by the Institutional Animal Care and Use Committee of NIBIOHN (approval ID: DSR02-24R3). All experiments with huACE2 Tg mice infected with SARS-CoV-2 were performed in enhanced BSL3 containment laboratories at the Tsukuba Primate Research Center of the NIBIOHN, following the approved standard operating procedures of the BSL3 facility.

### Purification of viral RNA and RT-qPCR

huACE2 Tg mice were assigned randomly to two groups (PBS-treatment (*n* = 5) and TP17/86 cocktail-treatment (*n* = 5)) to assess the viral load of SARS-CoV-2. Viral infection and administration of the TP17/86 cocktail were conducted as same as above. To measure the viral load of SARS-CoV-2 Tokyo strain in the lung, organs were homogenized in 3 ml of PBS using gentleMACS^TM^ Dissociator and M tubes (Miltenyi Biotec, Bergisch Gladbach, Germany). The lung RNAs were purified using 250 ul of lung lysate by TRIzol LS Reagent (Thermo Fisher Scientific, Waltham, MA, USA) according to the manufacturer’s protocol. Reverse transcriptase (RT) reactions were performed with ReverTra Ace qPCR RT master mix with gDNA remover (TOYOBO, Osaka, Japan) using 500 ng of lung RNA. To quantify the SRAS-CoV-2 subgenomic RNA, the RT reaction products were diluted with 1/10 and 5 μl of the diluents were subjected to quantitative real-time PCR using THUNDERBIRD Probe qPCR Mix (TOYOBO) and primer/probe sets as follows; 5′-CGATCTCTTGTAGATCTGTTCTC-3′ (forward primer), 5′-ATATTGCAGCAGTACGCACACA-3′ (reverse primer), and FAM-5′-ACACTAGCCATCCTTACTGCGCTTCG-3′-BHQ1 (probe). The qPCR conditions were 95 °C for 5 min, and 45 cycles of 15 s at 95 °C followed by 60 s at 60 °C. To examine the copy number of subgenomic RNA, PCR fragments amplified the same primer set as RT-qPCR were cloned into pMD vector and used for standards of RT-qPCR. To quantify the copy number of subgenomic RNA in the lung (*y*), copy number obtained from RT-PCR (*a*) was calculated as follows:

$${{y}}=\, 	 {{a}}\times \frac{3000\,({{{{{{\rm{total}}}}}}}\,{{{{{{\rm{lung}}}}}}}\,{{{{{{\rm{lysate}}}}}}})}{250\,({{{{{{\rm{lysate}}}}}}}\,{{{{{{\rm{for}}}}}}}\,{{{{{{\rm{RNA}}}}}}}\,{{{{{{\rm{extraction}}}}}}})} \times \frac{({{{{{{\rm{total}}}}}}}\,{{{{{{\rm{RNA}}}}}}}\,{{{{{{\rm{in}}}}}}}\,0.25\,{{{{{{\rm{ml}}}}}}}\,{{{{{{\rm{of}}}}}}}\,{{{{{{\rm{lysate}}}}}}})}{500\,({{{{{{\rm{RNA}}}}}}}\,{{{{{{\rm{for}}}}}}}\,{{{{{{\rm{RT}}}}}}}\,{{{{{{\rm{reaction}}}}}}})} \\ 	 \times \frac{100\,({{{{{{\rm{total}}}}}}}\,{{{{{{\rm{RT}}}}}}}\,{{{{{{\rm{reaction}}}}}}})}{5\,({{{{{{\rm{RT}}}}}}}\,{{{{{{\rm{reaction}}}}}}}\,{{{{{{\rm{for}}}}}}}\,{{{{{{\rm{RT}}}}}}}-{{{{{{\rm{qPCR}}}}}}})}$$

Original data are available in Supplementary Data.

### Statistical analyses

Statistical analyses were performed by GraphPad Prism 7.0f (GraphPad Software, La Jolla, CA, USA). The Student’s two-tailed *t* test was used for body weight and RT-qPCR of subgenomic RNA, and the log-rung test was used for survival rate. *p* < 0.05 was regarded as statistically significant.

### Reporting summary

Further information on research design is available in the Nature Portfolio Reporting Summary linked to this article.

## Results

**Fig. 1:**
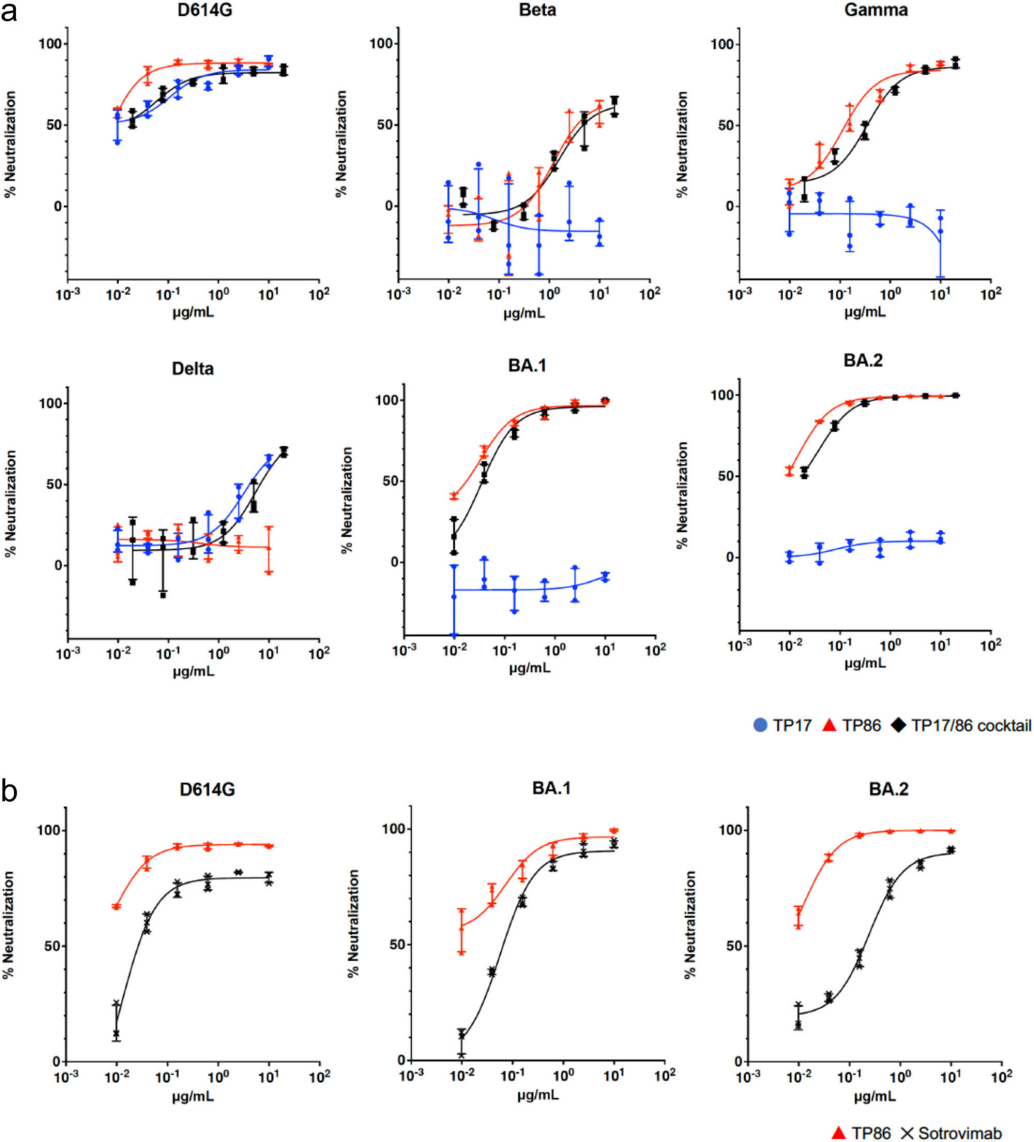
Neutralization of SARS-COV-2 VOCs by trimerized nanobodies. **a** Neutralization curves of the indicated SARS-COV-2 variants in HOS-ACE2-TMPRSS2 cells by trimerized nanobodies, TP17, TP86 and the TP17/86 cocktail. **b** Neutralization curves of the indicated SARS-COV-2 variants by TP86 and sotrovimab. Data are presented as the average ± SD (*n* = 3).

We modified P17 and P86 into dimers, named DP17 and DP86, and trimers, named TP17 and TP86 (Supplementary Fig 1), and found that trimers showed higher neutralizing activity against SARS-CoV-2 VOCs (Supplementary Fig. 2). We next examined neutralizing activity of TP17 and TP86 against VOCs including the Omicron variants BA.1 and BA.2. As expected from our previous dimer experiments, both TP17 and TP86 potently neutralized the original D614G (IC_50_ = 0.11 and <0.01 μg/ml, respectively, Table 1). TP86 potently neutralized Beta and Gamma (IC_50_ = 1.2 and 0.11 μg/ml, respectively), but not Delta, while TP17 neutralized Delta (IC_50_ = 3.2 μg/ml), but not Beta nor Gamma (Fig. 1a and Table 1). Although the two Omicron variants share 21 mutations in the spike protein, each variant has specific mutations, 13 in BA.1 and 8 in BA.2, which should be attributable to their different immune evasion profiles. BA.1 is highly resistant to most clinically available human monoclonal antibodies except for sotrovimab^3,4^ Sotrovimab efficiently neutralized D614G, BA.1 and BA.2 (Fig. 1b, IC_50_ = 0.012, 0.057 and 0.24, respectively). TP86 neutralized BA.1 more potently than sotrovimab (Fig. 1b). BA.2 was recently reported to be highly resistant to even sotrovimab, 27.049.7 folds more resistant than the original strain^16,17^. Our pseudovirus infectivity assays also showed that BA.2 is highly resistant to sotrovimab; the IC_50_ value is 20-fold higher than that of D614G. Strikingly, our TP86 neutralized BA.2 as potently as it neutralized BA.1, with IC_50_ = < 0.01 μg/ml (Fig. 1b).

**Table 1:** IC50 of nanobody clones (μg/ml).

					Omicron	
	D614G	Beta	Gamma	Delta	BA.1	BA.2
TP17	0.11	>10	>10	3.2	>10	>10
TP86	0.0051	1.2	0.11	>10	0.033	0.014
TP 17 + TP86	0.059	1.6	0.35	5.5	0.035	0.038

We also tested the cocktail of TP17 and TP86 (TP17/86 cocktail) to confirm whether the mixture of the two nanobodies could also neutralize VOCs. We found that the TP17/86 cocktail efficiently suppressed the infectivity of pseudoviruses bearing spikes of VOCs including the Omicron variants (Fig. 1a; black line). These results indicate that the TP17/86 cocktail could neutralize all existing VOCs.

**Fig. 2:**
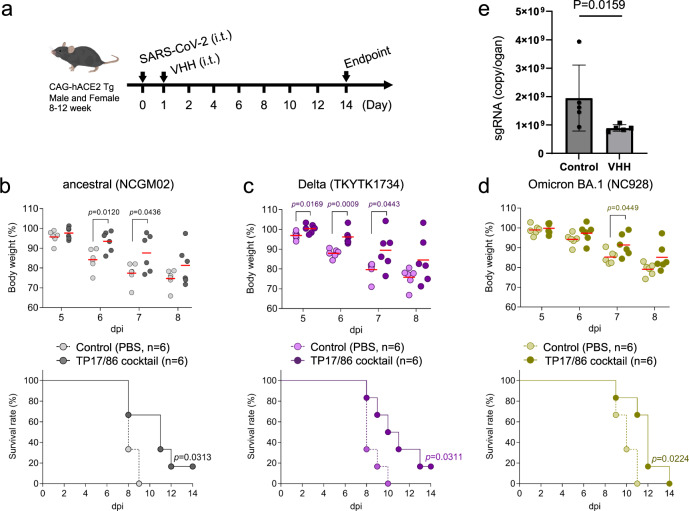
The TP17/86 cocktail delays SARS-CoV-2 infection in huACE2 Tg mice. **a** Overview of the experiment design. Lethal dose of SARS-CoV-2 (ancestral strain and Delta, 1 × 10^4^ TCID_50_; Omicron, 1 × 10^5^ TCID_50_) was intratracheally (i.t.) inoculated to six mice (3 male and 3 females) and followed by the administration of the TP17/86 cocktail (dark colors) or vehicle (bright colors) at dpi 1. The TP17/86 cocktail suppressed body weight loss and prolonged survival of the mice infected with ancestral strain (**b**), Delta (**c**), and Omicron BA.1 (**d**). **e** Subgenomic RNA levels of SARS-CoV-2 Tokyo strain at dpi 3. Data are represented as the average and ± SD (*n* = 5).

To evaluate the therapeutic potential of the TP17/86 cocktail for the treatment of VOCs including Omicron, we tested the nanobody cocktail using transgenic mice expressing human ACE2 (huACE2 Tg mice). These mice are highly susceptible to SARS-CoV-2 infection and show body weight loss reflecting disease severity^14^. The mice were infected with lethal doses of SARS-CoV-2 strains, TCID_50_ 1 × 10^4^ for the ancestral strain (SARS-CoV-2/UT-NCGM02/Human/2020/Tokyo; NCGM02^18^) and Delta (TKYTK1734^15^), and TCID_50_ 1 × 10^5^ for Omicron (hCoV-19/Japan/NC928-2N/2021; NC928^13^), and died 10–14 days post infection (dpi). We then treated them with a single dose of the TP17/86 cocktail intratracheally (TP17 0.6 mg/kg, TP86 0.6 mg/kg) on the next day following the infection (Fig. 2a). The intratracheal administration of TP17/86 cocktail significantly suppressed weight loss and prolonged survival of the mice infected with the ancestral strain, as expected (Fig. 2b) as well as those with the more pathogenic strain Delta, which is sensitive to TP17 in vitro (Fig. 2c). We also tested the efficacy of the TP17/86 cocktail against Omicron BA.1. The TP17/86 cocktail clearly suppressed weight loss and prolonged survival of the mice challenged with Omicron BA.1 (Fig. 2d). Finally, we measured viral load of SARS-CoV-2 Tokyo strain in the lung tissue at 3 dpi, and found that the TP17/86 cocktail significantly suppressed subgenomic RNA levels (Fig. 2e). These data demonstrate the in vivo therapeutic efficacy of the TP17/86 cocktail against SARS-CoV-2 VOCs.

## Discussion

The emergence and spread of the Omicron variants are apparently ongoing world health concerns. The immune evasion of Omicron variants makes it difficult to prevent and treat them while their pathogenesis and characteristics are still under investigation. Urgent steps we can take include elucidating the efficacy of available vaccines and intensifying their effects by combination and/or booster immunization^19,20^. Another step is to develop novel antibodies effective against Omicron variants, as antibody therapy is now proved highly effective for SARS-CoV-2 infection. In this study, we first demonstrate that nanobody TP86 potently inhibits the infectivity of Omicron variants BA.1 and BA.2. This is intriguing because these variants are highly resistant to most of clinically available human antibodies and BA.2 is resistant to even sotrovimab. Second, the TP17/86 cocktail potently suppressed in vitro all the VOCs reported so far, suggesting it has a broadly neutralizing activity. Third, intratracheal administration of our TP17/86 cocktail suppressed weight loss and prolonged survival of human ACE2 transgenic mice that were infected with lethal dose of SARS-CoV-2 including Omicron variant. It is difficult to translate these findings directly to human clinical settings, but our strategy to administer nanobodies intratracheally could be used for the treatment of COVID-19 patients or post exposure prophylaxis for severe disease development in the future. Further animal studies and preclinical studies will be needed to prove this hypothesis.

## Supplementary information

Supplementary Information

Supplementary Data

Description of Additional Supplementary Files

Reporting Summary

## Data Availability

The source data underlying Figs. 1 and 2 is available in the Supplementary Data file.
