# Supplementary material for: Intratracheal trimerized nanobody cocktail administration suppresses weight loss and prolongs survival of SARS-CoV-2 infected mice
Source: Commun Med (Lond). 2022 Nov 26;2:152. doi: 10.1038/s43856-022-00213-5 (PMC9701191; doi:10.1038/s43856-022-00213-5)
Supplement: Supplementary file 1 — Supplementary Information [file 43856_2022_213_MOESM1_ESM.pdf]

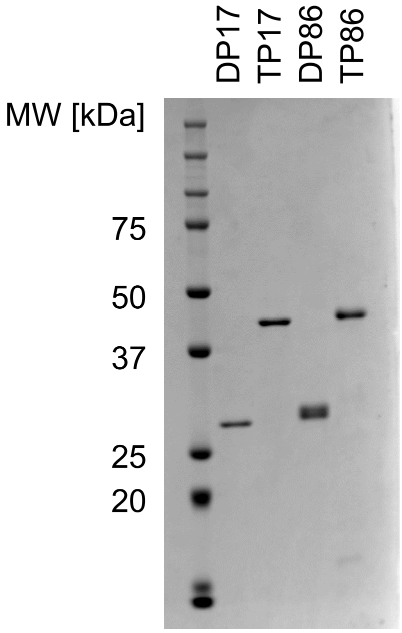

Supplementary Figure 1 CBB staining of nanobody dimers and trimers.

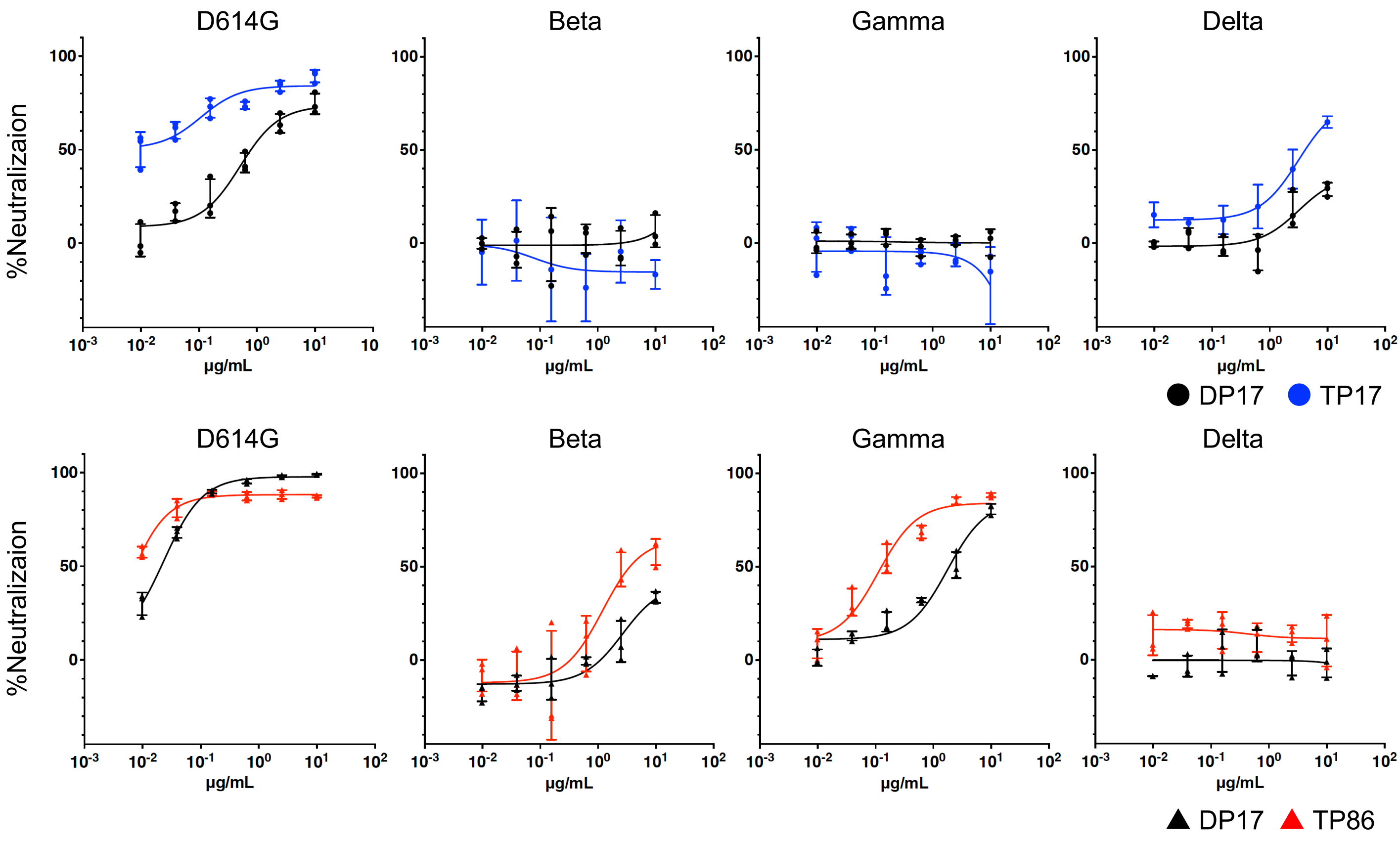

Supplementary Figure 2 Neutralization of SARS-CoV-2 VOCs by dimerized and trimerized nanobodies.
