# Supplementary material for: Intratracheal trimerized nanobody cocktail administration suppresses weight loss and prolongs survival of SARS-CoV-2 infected mice
Source: Commun Med (Lond). 2022 Nov 26;2:152. doi: 10.1038/s43856-022-00213-5 (PMC9701191; doi:10.1038/s43856-022-00213-5)
Supplement: Supplementary file 3 — Description of Additional Supplementary Files [file 43856_2022_213_MOESM3_ESM.pdf]

Description of Additional Supplementary Files

File Name: Supplementary Data

Description: Original data for each figure
